# Overhauling the Effect of Surface Sterilization on Analysis of Endophytes in Tea Plants

**DOI:** 10.3389/fpls.2022.849658

**Published:** 2022-05-03

**Authors:** Yueer Yu, Zimeng Chen, Hengtong Xie, Xiaoxiao Feng, Yuefei Wang, Ping Xu

**Affiliations:** ^1^Institute of Tea Science, Zhejiang University, Hangzhou, China; ^2^Agricultural Experiment Station, Zhejiang University, Hangzhou, China; ^3^Key Laboratory of Horticultural Plant Growth, Development and Quality Improvement, Ministry of Agriculture, Hangzhou, China

**Keywords:** endophytes, surface sterilization, *Camellia sinensis*, sodium hypochlorite, diversity

## Abstract

Increasing evidence shows that plant Endophytes play a crucial role in the fitness and productivity of hosts. Surface sterilization is an indispensable process before high-throughput sequencing (HTS) and tissue separation of plant endophytes, but its potential impact on the composition and diversity of endophytes has rarely been investigated. In the present work, the influence of sodium hypochlorite (NaClO) on the diversity of endophytic bacteria and fungi in leaves and stems of tea plants was investigated. We found that the diversity of bacterial endophytes was significantly affected by the concentration of NaClO as well as the pretreatment time. Pretreatment with 0.5% NaClO for 8 min and 2.0% NaClO for 3 min were suitable for the tea plant leaves and stems, respectively, but the effects of NaClO on the diversity of fungal endophytes were limited according to the results from HTS. Regardless of NaClO sterilization, most of the endophytes in tissues, such as the dominant taxa, could not be Isolated by using the regular culture-dependent approaches. Collectively, our results demonstrated that the pretreatment with NaClO should be modified to precisely understand the diversity of endophytes from different tissues of tea plants and also indicate that more attention should be paid to establish specific culture-dependent protocols for the isolation of plant endophytes.

## Introduction

As microorganisms inhabiting internal tissue of plants with no negative impacts on hosts, endophytes, the important components of plant microbiomes (Porras-Alfaro and Bayman, 2011), have attracted more attention around the world in the last few decades (White et al., 2019). The colossal biological diversity and the potential for biosynthesizing phytochemicals have made endophytes microbial resources with great research value and prospects (Jamwal and Gandhi, 2019). In terms of the symbiotic relationship, endophytes exert positive effects on the host plant in the following ways: (1) Modulating the growth of the host (Saravanan et al., 2008; Shi et al., 2010; Beltran-Garcia et al., 2014; Verma et al., 2017); (2) Enhancing the abiotic and biotic stress tolerance of hosts (Redman et al., 2002; Irizarry and White, 2018; Zhang et al., 2019); (3) Influencing the production of secondary metabolites in the host (Koskimäki et al., 2009; Verginer et al., 2010); and (4) Increasing the resistance of the host against pathogens and pests (Arnold et al., 2003; Hartley and Gange, 2009; Gond et al., 2015; Busby et al., 2016; Xie et al., 2020). From an individual perspective, the various secondary metabolites produced by endophytes not only enrich the chemical diversity of biologically active molecules, but also make the endophytes a bioengineering tool for drug discovery (Alvin et al., 2014; Li et al., 2018; Sarsaiya et al., 2019).

As a worldwide economic woody crop, tea plants (*Camellia sinensis*) play essential roles in the forest ecosystem in which endophytic microorganisms have been a research focus. According to previous reports, endophytic fungi isolated from tea plants cover 3 phyla, 5 classes, 14 orders, 24 families, and 34 genera, and endophytic bacteria include 4 phyla, 7 classes, 13 orders, 24 families, and 32 genera (Xie et al., 2020). Research on biological functions of tea plant endophytes has found that some tea endophytes not only show antagonism toward tea plant pathogens (Rabha et al., 2014), but they also have antagonistic effects on other plant pathogens. In addition, endophytes have the capacity to promote the growth of tea plants (Nath et al., 2015; Yan et al., 2018; Borah et al., 2019) as well as to produce or modify tea plant metabolites (Agusta et al., 2005; Wang et al., 2014; Sun et al., 2019).

The previous research has shown that endophytes isolated from tea plants are worth studying in depth. The most frequently utilized method to isolate endophytes is the tissue separation with surface sterilization, and the factors of surface sterilization have a significant influence on the results. Theoretically, the only condition to determine the exposure duration is complete surface sterilization. However, because of the agents’ penetration, it is difficult to eliminate the influence agents have on endophytic diversity and composition (Hallmann et al., 2006). Underexposure to the agent leads to the contamination of cultivable microorganisms and amplifiable nucleic acid, but overexposure may cause damage to the endophytes (Lundberg et al., 2012) by inducing DNA mutations (Tosi et al., 2021). Therefore, it is of importance to established a specific protocol for endophyte isolation from tea plants.

This study was set out to make a thorough exploration in the influence of the surface sterilization method based on the sodium hypochlorite (NaClO), the most commonly used sterilizing agent (Tosi et al., 2021), on diversity and composition of tea plants’ foliar and cauline endophytes by comprehensively analyzing the results from high-throughput sequencing (HTS) and tissue separation and then to find an efficient and specific endophytic microorganism isolation method for *Clonorchis sinensis*.

## Materials and Methods

### Sample Collection and Preprocessing

Symptomless mature leaves and stems with no disease or damage were collected from two varieties of *Camellia sinensis*, such as cultivar Longjing 43, from Yuhang county, Zhejiang Province, China, and cultivar Jiukeng, from Chun’an County, Zhejiang Province, China. All samples were placed under running tap water to remove adherent dust and soil particles and most microbial Surface impurities before further processing (Hallmann et al., 2006).

### Sterility Examination of Surface Sterilization Combinations

The surface sterilization method was based on five concentrations of NaClO (0.25, 0.5, 1.0, 2.0, and 4.0%) and different durations for tissue immersion in the agent (1–15 min) (Xie et al., 2020). After the pretreatment, samples were sterilized with different combinations, followed by rinsing with sterile water three times. The last rinse was taken at the scale of 100 μl/dish onto Luria-Bertani (LB) medium and potato dextrose agar (PDA) medium, and samples were incubated in dark at 30°C for 7 days to confirm whether sterilization was complete.

### DNA Extraction and High-Throughput Sequencing

However, the agent may permeate into the tissue and create conditions lethal for some endophytic microorganisms (Hallmann et al., 2006), HTS was employed to study the influence of the agent on Endophytes. DNA was extracted from the surface-sterilized samples according to the ALFA-SEQ Plant DNA Kit (mCHIP, China) and concentration and purity of which were determined by using the NanoDrop One (Thermo Scientific, Wilmington, United States). The V4 region of the Bacterial 16S rRNA genes was amplified by using 515f (5′ GTGYCAGCMGCCGCGGTAA 3′)/806r (5′ GGACTACNVGGGTWTCTAAT 3′) primers (Parada et al., 2016; Liu et al., 2018; Matsumoto et al., 2021), and the blocking primers peptide nucleic acids (mPNA and pPNA) were added to prevent the amplification of mitochondrial and chloroplast DNA (Lundberg et al., 2013). The ITS hypervariable region was amplified by using ITS1f (5′ CTTGGTCATTTAGAGGAAGTAA) and ITS2r (5′ GCTGCGTTCTTCATCGATGC) (Nilsson et al., 2019). For 16S PCR amplification, the thermocycler program was set for initial denaturing at 95°C for 5 min, followed by 30 cycles of denaturing at 96°C for 1 min, PNA annealing at 78°C for 5 s, primer annealing at 54°C for 1 min, extension at 74°C for 1 min, and a final extension at 74°C for 10 min. The ITS PCR procedures were as follows: pre-denaturation at 95°C for 5 min, followed by 30 cycles of deformation at 94°C for 30 s, annealing at 52°C for 30 s, elongation at 72°C for 30 s, and a final extension at 72°C for 10 min. The PCR products were extracted on 1.0% agarose gels, the concentrations of which were examined by the GeneTools Analysis Software (Version 4.03.05.0, SynGene). The fragments purified with an E.Z.N.A. Gel Extraction Kit (Omega, United States) were sequenced under by PE250 based on the Illumina Nova 6000 platform (Illumina, San Diego, United States) at Guangdong Magigene Biotechnology Co., Ltd., (Guangzhou, China).

### Isolation of Endophytic Fungi by Tissue Separation

The surface-sterilized samples were cut aseptically into segments (2 mm × 2 mm for leaf tissue (Liu et al., 2015) and 3 mm for debarked stem samples (Win, 2018)) with a sterilized blade before being plated on malt extract agar (MEA) medium and PDA medium in 9-cm diameter plastic Petri dishes (10 segments per Petri dish). The Petri dishes were incubated at 26°C in dark for 7 days. Each isolate was inoculated on corresponding medium in 6-cm diameter plastic Petri dishes for pure culture and further processing.

### Isolation of Endophytic Bacteria by Tissue Separation

After the surface sterilization, samples were homogenized at the ratio of 20 ml sterile water to 6 g leaf tissue and 15 ml sterile water to 6 g stem tissue, followed by the graded dilution to 10^–1^, 10^–2^, and 10^–3^ with sterilized water (Yan et al., 2018). Then, 200 μl diluted homogenate was removed and spread on the LB agar medium and nutrient agar (NA) medium in 9-cm diameter Petri dishes. The Petri dishes were placed in the incubator at 30°C and in the dark for 5 days. The streak plate method was used for the purification.

### Molecular Identification of Isolated Endophytes

The sequence analysis of the 16S rRNA gene and the ITS rDNA region was used for the species-level identification of isolated endophytic bacteria and fungi, respectively. The ITS region was amplified with primers of ITS1 (5′ TCCGTAGGTGAACCTGCGG)/ITS4 (5′ TCCTCCGCTTATTGATATGC) (Pryce et al., 2003; Kehelpannala et al., 2018) and 16s rRNA genes were amplified with primers of 27f (5′ AGAGTTTGATCMTGGCTCAG)/1492r (5′ ACGGTTACCTTGTTACGACTT) (Wei et al., 2018). The sequences of isolates were subjected to a BLAST search^1^ and compared with representative sequences in the NCBI database to determine the corresponding species (Vu et al., 2019).

### High-Throughput Sequencing Analysis

The raw reads transformed from original data were quality-filtered by FASTP (version 0.14.1.). The paired-end reads were processed using the USEARCH (version 10.0.240). The operational taxonomic units (OTUs) that had a 97% similarity level using UPARSE (Edgar, 2013) were processed by utilizing QIIME2 (version 2020.11.0). The taxonomy of each 16S rRNA gene sequence was analyzed against the SILVA (v123) 16S rRNA gene database at a confidence threshold of 80%. The taxonomy of each ITS rRNA gene sequence was analyzed against the RDP (v2) ITS rRNA gene database at a confidence threshold of 80%.

The alpha diversity based on the R vegan package was conducted to reveal the within-habitat diversity and principal component analysis (PCA) was performed to examine dissimilarities in the community composition among samples based on an Euclidean metric. Venn diagrams showed the number of common or unique OTUs in multiple groups.

### Statistical Analysis

The statistical analysis and graphic illustration were performed using the GraphPad Prism version 9.0 (GraphPad Software Inc., San Diego, CA, United States) and SPSS Statistics version 26.0 (IBM Corporation., Armonk, NY, United States). Results were expressed as mean ± standard error of the mean (SEM). The statistical significance was indicated by one-way analysis of variance (ANOVA) followed by the Tukey’s multiple comparison test. A *p*-value less than 0.05 (*p* < 0.05) was considered statistically significant and the statistical differences are indicated by superscripts.

## Results

### Preliminary Screening of Surface Sterilization Methods

**FIGURE 1:**
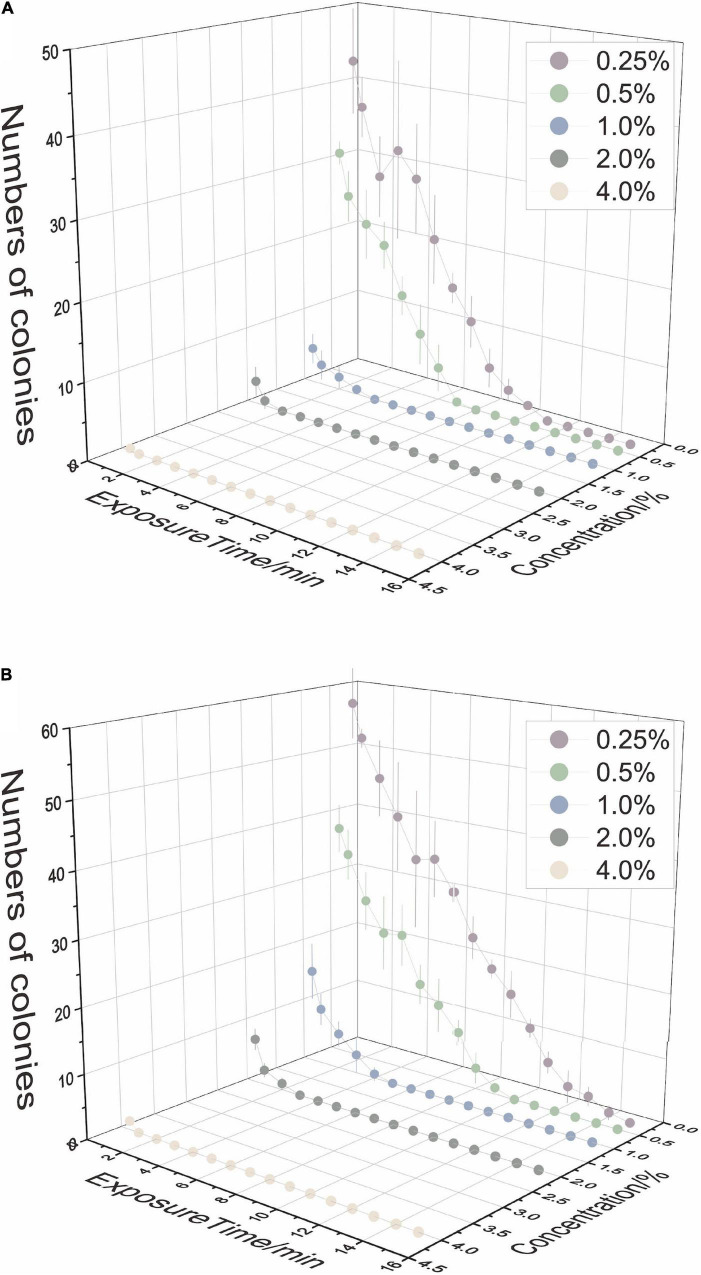
The disinfection ability of sodium hypochlorite (NaClO) concentrations and exposure time on tea plant leaves **(A)** and stems **(B)**.

The sterilization efficiency of each combination was shown by the number of microbes after 7-day incubation (Figure 1). To achieve complete surface sterilization, the lower the concentration of NaClO used, the longer the effect time needed; conversely, when using an agent with higher concentration, the reaction time could be slightly shorter. Meanwhile, the sterilization efficiency was different in leaf tissue and stem tissue. Mature stem tissue required a higher concentration or longer duration. The results showed that the environmental bacteria of tea plants were more plentiful than fungi, and their survival appeared to be more related to concentration than duration. Considering both efficiency and feasibility, three combinations of each kind of tissue were screened for further study: for leaf tissue, 0.5% NaClO for 8 min (LL), 1.0% NaClO for 4 min (ML), and 2.0% NaClO for 2 min (HL); for stem tissue, 0.5% NaClO for 10 min (LS), 1.0% NaClO for 5 min (MS), and 2.0% NaClO for 3 min (HS).

### Effects of Surface Sterilization on Endophytic Diversity

#### Diversity Analysis of Fungal Endophytes

**FIGURE 2:**
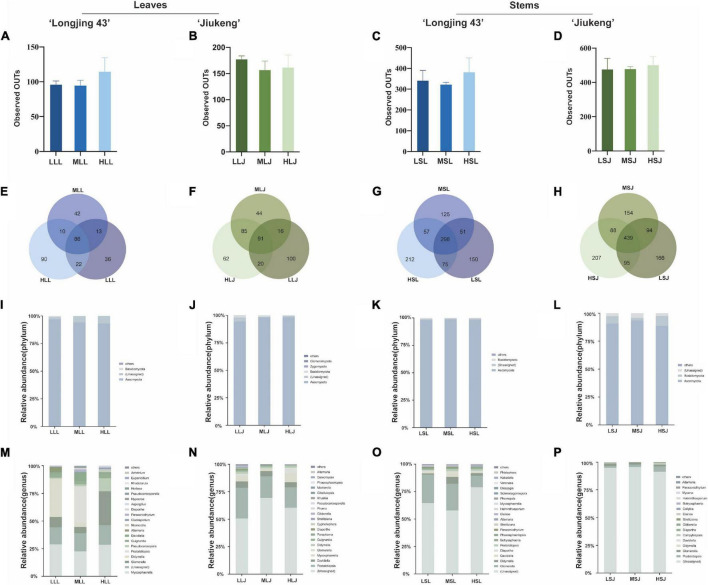
Effects of sterilization treatments on the diversity of fungal endophytes from tea plant leaves and stems. **(A–D)** Alpha diversity of the fungal endophytic community; **(E–H)** Venn diagram showing the number of shared and unique operational taxonomic units (OTUs) (at 97% similarity); **(I–L)** relative abundance of fungal endophytes at the phylum level; **(M–P)** relative abundance of fungal endophytes at the genus level. LLL, MLL, and HLL represent the leaf samples from cultivar Longjing 43 treated with 0.5, 1.0, and 2.0% of NaClO. LLJ, MLJ, and HLJ represent the leaf samples from cultivar Jiukeng treated with 0.5, 1.0, and 2.0% of NaClO. LSL, MSL, and HSL represent the stem samples from cultivar Longjing 43 treated with 0.5, 1.0, and 2.0% NaClO. LSJ, MSJ, and HSJ represent the leaf samples from cultivar Jiukeng treated with 0.5, 1.0, and 2.0% NaClO.

After removing chimeric, plastids, and mitochondrial sequences, raw reads were drawn flat by minimum sequence. There were 104,605 reads per leaf sample and 184,954 reads per stem sample from cultivar Longjing 43, 394,335 reads per leaf sample and 390,126 reads per stem sample from cultivar Jiukeng. The alpha diversity (Figures 2A–D) index (observed OTUs) revealed that the differences were not significant (analysis of significant differences in two groups is shown in Supplementary Table 1), indicating that the within-habitat diversity of fungal endophytes was not noticeably influenced by the surface sterilization methods. Venn diagrams (Figures 2E–H) of fungal endophytic OTUs displayed the number of shared and unique OTUs in samples treated with different surface sterilization methods. The numbers of OTUs for leaf samples ranged from 151 to 258, and for stem samples ranged from 531 to 829. Among them, the ratios of shared OTUs were 86 (28.76%) in leaf samples of cultivar Longjing 43, 91 (21.77%) in leaf samples of cultivar Jiukeng, 298 (30.79%) in stem samples of cultivar Longjing 43, and 439 (35.32%) in stem samples of cultivar Jiukeng. Each group had unique OTUs, the differences of which indicated the sterilization methods had a significant impact on Fungal endophyte composition.

Analysis at phylum level (Figures 2I–L) showed high proportions of Ascomycota (93.0–98.3%) for fungal endophytes in leaves. Ascomycota (88.6–98.5%) was dominant and Basidiomycota (0.52–8.9%) was abundant for fungal endophytes in stems. The proportion of Basidiomycota in leaf samples declined as the concentrations increased. In two cultivars’ leaves, (Figures 2M,O) common genera showed the same reaction to different surface sterilization methods: both *Mycosphaerella* and *Colletotrichum* had the lowest content in medium concentration; *Didymella, Pestalotiopsis*, *Diaporthe*, and *Pseudocercosporella* content were reduced by high concentrations. In the stems, (Figures 2N,P) *Glomerella* decreased sequentially with increasing concentrations; *Pestalotiopsis*, in contrast to *Helminthosporium*, was affected most by low concentration. Compared with consistency of endophytic bacteria in leaves and stems, trends in the common fungi in two tissue types were more uniform: *Davidiella*, *Pestalotiopsis*, *Didymella*, and *Diaporthe* had the highest percentages in medium concentration and *Glomerella* declined as dose increased in cultivar Longjing 43.

#### Diversity Analysis of Bacterial Endophytes

**FIGURE 3:**
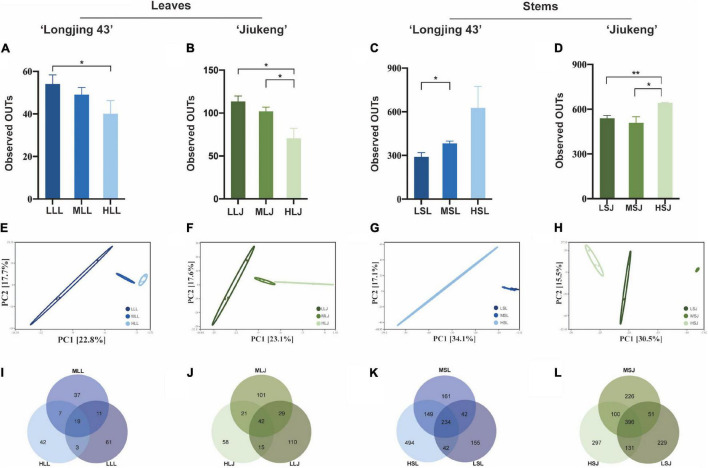
Effects of sterilization treatments on the diversity of bacterial endophytes from tea plant leaves and stems. **(A–D)** Alpha diversity of the bacterial endophytic community; **(E–H)** principal component analysis (PCA) of the bacterial community structure; **(I–L)** Venn diagram showing the number of shared and unique OTUs (at 97% similarity). LLL, MLL, and HLL represent the leaf samples from cultivar Longjing 43 treated with 0.5, 1.0, and 2.0% of NaClO. LLJ, MLJ, and HLJ represent the leaf samples from the cultivar Jiukeng treated with 0.5, 1.0, and 2.0% of NaClO. LSL, MSL, and HSL represent the stem samples from the cultivar Longjing 43 treated with 0.5, 1.0, and 2.0% NaClO. LSJ, MSJ, and HSJ represent the leaf samples from the cultivar Jiukeng treated with 0.5, 1.0, and 2.0% NaClO. Asterisks indicate significant differences (*p < 0.05 and **p < 0.01).

After qualified filtering the raw reads and normalizing the read counts, there were 3,945 reads per leaf sample and 55,804 reads per stem sample from cultivar Longjing 43, and 6,811 reads per leaf sample and 36,446 reads per stem sample from cultivar Jiukeng were retained. The alpha diversity (Figures 3A–D) index (observed OTUS) showed that the within-habitat diversity declined when the concentration was increased, indicating that the diversity of bacterial endophytes in leaves is influenced by the concentration of the agent. The results were opposite in stem samples in that the group treated with the highest concentration for the lowest exposed duration maintained the best richness. Bacterial endophytes in stem tissue were better tolerant of high concentrations compared to those in leaf tissue. Beta diversity analysis revealed clustering of differently treated samples when calculating PCA plots based on Euclidean distances (Figures 3E–H) and PCoA plots based on Bray–Curtis distances (Supplementary Figure 1). Venn diagrams (Figures 3I–L) of bacteria endophytic OTUs in samples of leaf tissue and stem tissue of each cultivar demonstrate the number of shared and unique OTUs in samples treated with different surface sterilization methods. At a 97% similarity level, the numbers of OTUs for leaf samples ranged from 49 to 196, and stem samples ranged from 473 to 924. The ratios of shared OTUs were not high in each group according to tissue types and cultivar types, respectively; there were 19 (10.56%) in leaf samples of cultivar Longjing 43 and 42 (11.48%) in leaf samples of cultivar Jiukeng. There were 234 (18.32%) in stem samples of cultivar Longjing 43 and 396 (27.69%) in stem samples of cultivar Jiukeng. In addition, each group had unique OTUs, the proportions of which indicated that, to some extent, differences in sterilization methods had an important impact on the bacterial endophyte composition.

**FIGURE 4:**
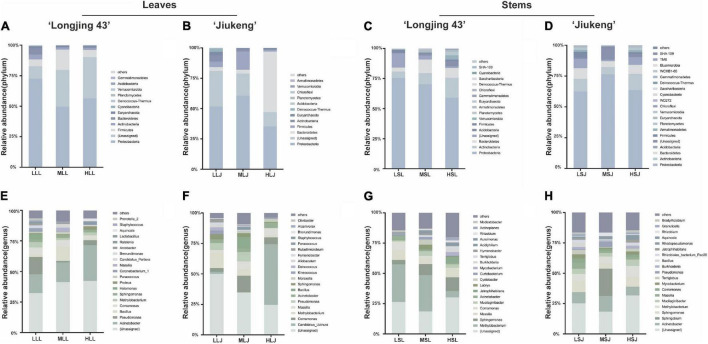
Effects of sterilization treatments on the composition of bacterial endophytes from tea plant leaves and stems at the phylum level **(A–D)** and genus level **(E–H)**. LLL, MLL, and HLL represent the leaf samples from the cultivar Longjing 43 treated with 0.5, 1.0, and 2.0% of NaClO. LLJ, MLJ, and HLJ represent the leaf samples from the cultivar Jiukeng treated with 0.5, 1.0, and 2.0% of NaClO. LSL, MSL, and HSL represent the stem samples from the cultivar Longjing 43 treated with 0.5, 1.0, and 2.0% NaClO. LSJ, MSJ, and HSJ represent the leaf samples from the cultivar Jiukeng treated with 0.5, 1.0, and 2.0% NaClO.

Bacterial endophytic structure and composition (Figure 4) showed that the abundant phyla (≥0.5% of all sequences across all samples) in leaves were Proteobacteria (35.2–72.5%), Firmicutes (1.4–5.9%), Bacteroidetes (2.8–49.9%), Actinobacteria (0.8–5.0%), and Euryarchaeota (0.6–2.9%). It was also observed that the abundant bacterial phyla in two cultivars showed similar trends under different treatments: *Firmicutes* showed the highest abundances in medium concentration treatment (ML) compared to the other two treatments; *Actinobacteria* and *Euryarchaeota* declined trend as the concentration increased. The common genera in two cultivars appeared to have same response to different treatments. Relative abundance of *Acinetobacter* increased while *Methylobacterium* decreased with a higher concentration agent. *Bacillus*, *Paracoccus*, and *Massilia* took up the highest proportion under the secondary dosage and processing time. In terms of the relative abundance of cauline bacterial endophytes, the dominant phyla (≥0.5% of all sequences across all samples) were Proteobacteria (59–76.4%), Actinobacteria (5.3–16.5%), Bacteroidetes (3.2–11.3%), and Firmicutes (0.6–4.6%). Actinobacteria had an advantage with 2.0% NaClO treatment. At the genus level, *Massilia* showed the same response, in that medium combination negatively affected the relative abundance. *Jatrophihabitans* and *Burkholderia* were identified as the most abundant among the three treatments in both cultivars. Although most endophytic bacteria had different characteristics in different tissue type, some of the common genera were found to have similarities: *Comamonas* in cultivar Longjing 43 showed intolerance to high concentration, *Pseudomonas* in cultivar Jiukeng accounted most in medium treatment group compared with other two groups and *Sphingomonas* was dominant in the low treatment group.

### Analysis of Cultured Endophytes

**FIGURE 5:**
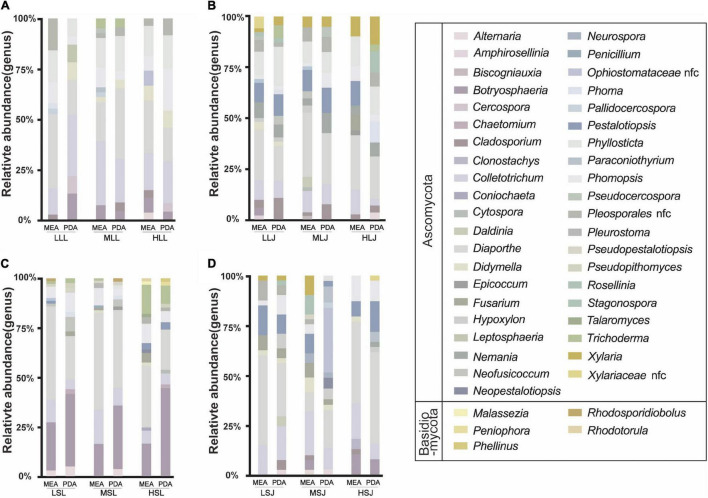
Effects of sterilization treatments on the composition of cultured fungal endophytes from tea plant leaves **(A,B)** and stems **(C,D)** in MEA and PDA medium. LLL, MLL, and HLL represent the leaf samples from the cultivar Longjing 43 treated with 0.5, 1.0, and 2.0% of NaClO. LLJ, MLJ, and HLJ represent the leaf samples from the cultivar Jiukeng treated with 0.5, 1.0, and 2.0% of NaClO. LSL, MSL, and HSL represent the stem samples from the cultivar Longjing 43 treated with 0.5, 1.0, and 2.0% NaClO. LSJ, MSJ, and HSJ represent the leaf samples from the cultivar Jiukeng treated with 0.5, 1.0, and 2.0% NaClO.

Endophytes isolated from tea plants are summarized in the Supplementary Table 2. There were 1,040 strains of fungal endophytes isolated in total, 43 strains per group on average, which were categorized by surface sterilization methods, cultivars, tissue types, and medium types. Two phyla and 46 genera were involved. As shown in Figure 5, statistics were consistent with the results of HTS at the phylum level; the proportion of Ascomycota (99.4%) was significantly higher than Basidiomycota (0.58%). At the genus level, non-conformity was found between the results of HTS and tissue isolation. Genera such as *Mycosphaerella* and *Davidiella*, which were abundant according to the results of HTS, were not isolated; *Diaporthe* accounted for up to 30.5% of isolated endophytic fungi but measured only 0.02–1.7% in the HTS results. However, *Botryosphaeria*, *Didymella*, *Alternaria*, and *Pestalotiopsis* were not only enriched in testing results, but also in abundance after isolation and cultivation. Based on a single factor comparison, medium types showed little influence on the diversity and quantity of cultivable genera, and MEA medium was slightly better than PDA medium. For leaf samples, treatment with 1.0% NaClO for 4 min (ML) gave better results than did the other two concentrations, while higher concentration combined with lower exposure time was more suitable for stem samples.

**FIGURE 6:**
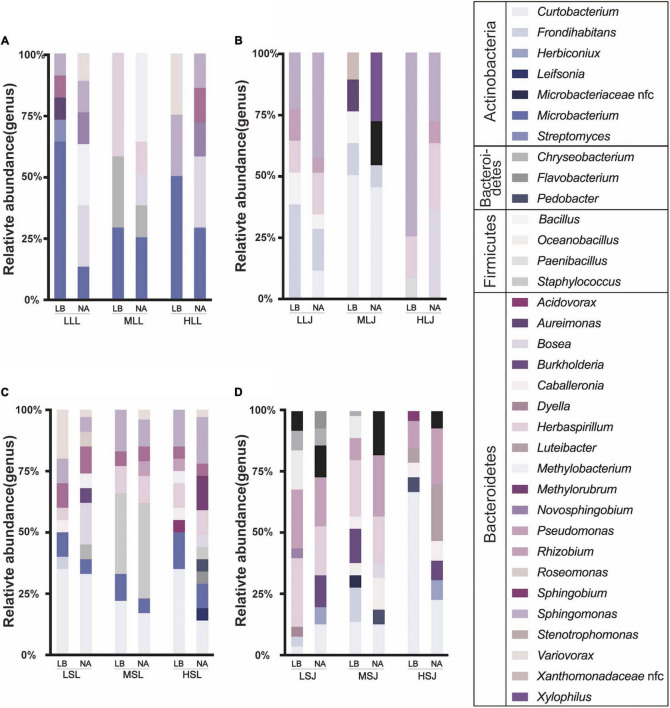
Effects of sterilization treatments on the composition of cultured bacterial endophytes from tea plant leaves **(A,B)** and stems **(C,D)** in LB and NA medium. LLL, MLL, and HLL represent the leaf samples from the cultivar Longjing 43 treated with 0.5, 1.0, and 2.0% of NaClO. LLJ, MLJ, and HLJ represent the leaf samples from the cultivar Jiukeng treated with 0.5, 1.0, and 2.0% of NaClO. LSL, MSL, and HSL represent the stem samples from the cultivar Longjing 43 treated with 0.5, 1.0, and 2.0% NaClO. LSJ, MSJ, and HSJ represent the leaf samples from the cultivar Jiukeng treated with 0.5, 1.0, and 2.0% NaClO.

There were 337 strains of bacterial endophytes isolated, covering four phyla and 34 genera (Figure 6). Proteobacteria (59.1%) and Actinobacteria (32.3%) were the dominant phyla, and Firmicutes and Bacteroidetes accounted for 6.2 and 2.4%, respectively. At the genera level, *Curtobacterium* (18.99%), *Sphingomonas* (15.73%), and *Herbaspirillum* (11.87%) were the top three with the highest percentages. Similar to fugal endophytes, statistical results at the genus level showed differences in cultivable ability and non-comformity between the results of HTS and tissue isolation. Relative consistency was maintained in sequencing and isolation in *Curtobacterium*, *Sphingomonas*, *Burkholderia*, and *Rhizobium*. The surface sterilization methods showed little influence on the diversity of isolation methods, and lower concentrations of the agent were more suitable for isolating the scarce strains. Meanwhile, endophytic bacteria species preferred tissue types in this study. For the diversity, cultivable bacterial endophytes from stems included 30 genera and 20 genera from leaf samples covered. *Bosea*, *Herbaspirillum*, and *Sphingomonas* were isolated from both leaf samples and stem samples, and all *Burkholderia* strains and *Caballeronia* strains were isolated from stem samples.

## Discussion

During the long period of co-evolution, a mutually beneficial relationship was gradually established between endophytes and their host plants (Saikkonen et al., 2004; Wang and Dai, 2011; Wani et al., 2015; Baltrus, 2017). The tea plant is one of the oldest domesticated plants in the world, and its endophytes play an important role in resisting biotic and abiotic stress (Rabha et al., 2014; Zhao et al., 2014), as well as participating in secondary metabolite production (Agusta et al., 2005). Surface sterilization is used to eliminate the non-endophytic microorganisms, which is essential for precisely exploring the endophytes (Shan et al., 2018; Jia et al., 2021). Here, we found that the diversity and composition of bacterial endophytes were influenced significantly by the NaClO concentration, while the diversity of fungal endophytes was relatively stable under higher concentrations of NaClO or a longer exposure time.

Consistent with the observations in previous studies (Fukuzaki, 2006; Hallmann et al., 2006; Gonzaga et al., 2015; Egamberdieva et al., 2017), NaClO showed similar dose-dependent and exposure time-dependent effects in our study. To figure out the detailed profiles underlying the effect of NaClO on endophytes in tea plants, the high-throughput sequencing was subsequently employed. Both diversity and composition of fungal endophytes were not significantly affected by the concentration of NaClO. More specifically, Ascomycota took up the most fungal endophytes while Proteobacteria, Bacteroidetes, and Actinobacteria had dominant proportions of bacterial endophytes. NaClO causes biosynthetic alterations in cellular metabolism and phospholipid destruction and hypochlorous acids (HClO-) present in the NaClO solutions may act as solvents in contact with organic tissue to release chlorine. Chlorine is a strong oxidant in solution that forms chloramines when combined with the protein amino group that then disrupt the cell metabolism and inhibit essential bacterial enzymes, leading to irreversible oxidation of SH groups (Estrela et al., 2002; Fukuzaki, 2006; Sahu et al., 2022). Bacteria may be more sensitive than fungi upon treatment with NaClO.

In addition to surface sterilization, the culture medium also has a vital effect on the isolation of endophytes. *Bacillales* of Bacilli was abundant in endophytes isolated and identified from tea plants (Xie et al., 2020), which was further confirmed by our results. Previously, the selective effect of mediums on the isolation of endophytic fungi from an Indian neem plant *Azadirachta indica A. Juss*. was investigated. This study found that not only the quantity, but also the variety and growth rate of endophytic fungi were affected by the culture medium, indicating media preference was one of the critical factors that can directly influence the endophytic isolation (Verma et al., 2011). Meanwhile, the flora preference of the cultivable endophytic bacteria influenced by the culture medium was also observed in *Dendrobium*, wheatgrass, and *Passiflora incarnata* (Ringelberg et al., 2012; Goulart et al., 2019; Wang et al., 2019). Furthermore, differences exist between endophytic bacteria in the results from HTS and those from culture-dependent methods, especially at the genus level (Wang et al., 2019). In our work, *Diaporthe* was found to the dominant genus in cultured endophytes, while *Mycosphaerella* and *Davidiella*, the most abundant in HTS results, were not cultured successfully. Similar results were also observed in endophytic bacteria when comparing the results from culture-dependent methods and HTS.

Moreover, the potential functions of endophytes from tea plants were previously studied. For instance, *Alternaria alternata*, a species from the genus *Alternaria*, was found to produce bioactive metabolites that could inhibit the pathogenic microorganisms (Wang et al., 2014). *Diaporthe* sp. isolated from tea plants could stereoselectively oxidize the C-4 carbon of two R-substituted flavans to a 3-hydroxy structure from the same direction (Agusta et al., 2005). *Bacillus* and *Pseudomonas* had positive effects in growth promotion and disease prevention (Chakraborty et al., 2006; Morang and Dutta, 2012). However, in the present work, *Staphylococcus* and *Bacillus*, belonging to the order of Bacillales, were found to be susceptible to high concentrations of NaClO, indicating that NaClO sterilization increases the false-negatives in exploration of the functional endophytes in tea plants, and the effect of NaClO on microbiome research should be studied more in the future.

## Conclusion

The diversity of bacterial endophytes in leaves and stems of tea plants was significantly affected by the concentration of NaClO as well as the sterilization time. In comparison, composition of fungal endophytes in tea tissues was less susceptible to NaClO. Thus, it is suggested that sterilization with NaClO should be modified to precisely understand the diversity of the bacterial endophytes from different tissues in tea plants.

## Data Availability Statement

The data presented in the study are deposited in the Sequence Read Archive of NCBI repository, accession number PRJNA794727.

## Author Contributions

PX designed the study. YY and ZC collected the samples. YY, ZC, HX, and XF performed the laboratory experiments and analyzed the data. YY wrote the manuscript. PX and YW critically reviewed the manuscript. All authors read and approved the final manuscript.

## Conflict of Interest

The authors declare that the research was conducted in the absence of any commercial or financial relationships that could be construed as a potential conflict of interest.

## Publisher’s Note

All claims expressed in this article are solely those of the authors and do not necessarily represent those of their affiliated organizations, or those of the publisher, the editors and the reviewers. Any product that may be evaluated in this article, or claim that may be made by its manufacturer, is not guaranteed or endorsed by the publisher.

## Footnotes

1
https://blast.ncbi.nlm.nih.gov/Blast.cgi
