# Supplementary material for: Overhauling the Effect of Surface Sterilization on Analysis of Endophytes in Tea Plants
Source: Front Plant Sci. 2022 May 3;13:849658. doi: 10.3389/fpls.2022.849658 (PMC9111953; doi:10.3389/fpls.2022.849658)
Supplement: Supplementary file 1 [file Data_Sheet_1.docx]

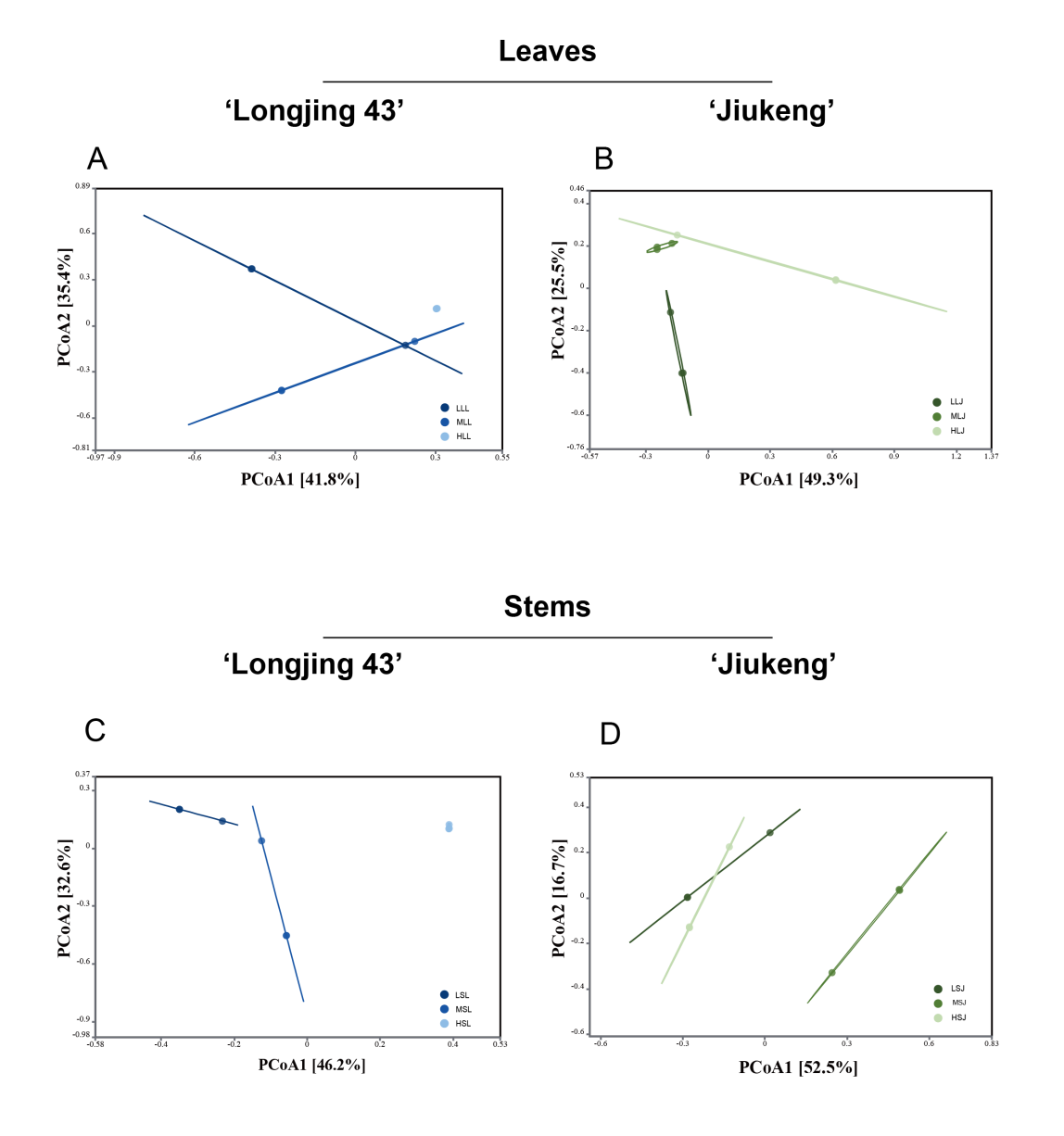


Figure S1. (A-D) Principal co-ordinates analysis (PCoA) of the bacterial community structure. LLL, MLL, HLL represented the leaf samples from ‘LongJing 43’ disposed with 0.5%, 1.0%, 2.0% of NaClO; LLJ, MLJ, HLJ represented the leaf samples from ‘JiuKeng’ disposed with 0.5%, 1.0%, 2.0% of NaClO; LSL, MSL, HSL represented the stem samples from ‘LongJing 43’ disposed with 0.5%, 1.0%, 2.0% of NaClO; LSJ, MSJ, HSJ represented the leaf samples from ‘JiuKeng’ disposed with 0.5%, 1.0%, 2.0% of NaClO.

Table S1 Analysis of two-groups significant difference of Observed OUTs index

| group1 | group2 | p-value |
| --- | --- | --- |
| HLL | LLL | 0.25 |
| HLL | MLL | 0.23 |
| LLL | MLL | 0.81 |
| HLJ | LLJ | 0.37 |
| HLJ | MLJ | 0.78 |
| LLJ | MLJ | 0.16 |
| HSL | LSL | 0.46 |
| HSL | MSL | 0.27 |
| LSL | MSL | 0.58 |
| HSJ | LSJ | 0.62 |
| HSJ | MSJ | 0.50 |
| LSJ | MSJ | 0.96 |

Table S2 Summary of isolated endophytes

| Kingdom | Phylum | Class | Order | Family | Genus |
| --- | --- | --- | --- | --- | --- |
| bacteria | Actinobacteria | Actinomycetia | Micrococcales | *Microbacteriaceae* | *Curtobacterium* |
|  |  |  |  |  | *Frondihabitans* |
|  |  |  |  |  | *Herbiconiux* |
|  |  |  |  |  | *Leifsonia* |
|  |  |  |  |  | *Microbacteriaceae* nfc |
|  |  |  |  |  | *Microbacterium* |
|  |  |  | Streptomycetales | *Streptomycetaceae* | *Streptomyces* |
|  | Bacteroidetes | Flavobacteriia | Flavobacteriales | *Weeksellaceae* | *Chryseobacterium* |
|  |  |  |  | *Flavobacteriaceae* | *Flavobacterium* |
|  |  | Sphingobacteriia | Sphingobacteriales | *Sphingobacteriaceae* | *Pedobacter* |
|  | Firmicutes | Bacilli | Bacillales | *Bacillaceae* | *Bacillus* |
|  |  |  |  |  | *Oceanobacillus* |
|  |  |  |  | *Paenibacillaceae* | *Paenibacillus* |
|  |  |  |  | *Staphylococcaceae* | *Staphylococcus* |
|  | Proteobacteria | Alphaproteobacteria | Hyphomicrobiales | *Aurantimonadaceae* | *Aureimonas* |
|  |  |  |  | *Boseaceae* | *Bosea* |
|  |  |  |  | *Methylobacteriaceae* | *Methylobacterium* |
|  |  |  |  |  | *Methylorubrum* |
|  |  |  |  | *Rhizobiaceae* | *Rhizobium* |
|  |  |  | Rhodospirillales | *Acetobacteraceae* | *Roseomonas* |
|  |  |  | Sphingomonadales | *Sphingomonadaceae* | *Novosphingobium* |
|  |  |  |  |  | *Sphingobium* |
|  |  |  |  |  | *Sphingomonas* |
|  |  | Betaproteobacteria | Burkholderiales | *-* | *Xylophilus* |
|  |  |  |  | *Burkholderiaceae* | *Burkholderia* |
|  |  |  |  |  | *Caballeronia* |
|  |  |  |  | *Comamonadaceae* | *Acidovorax* |
|  |  |  |  |  | *Variovorax* |
|  |  |  |  | *Oxalobacteraceae* | *Herbaspirillum* |
|  |  | Gammaproteobacteria | Xanthomonadales | *Rhodanobacteraceae* | *Dyella* |
|  |  |  |  |  | *Luteibacter* |
|  |  |  |  | *Xanthomonadaceae* | *Stenotrophomonas* |
|  |  |  |  |  | *Xanthomonadaceae* nfc |
|  |  |  | Pseudomonadales | *Pseudomonadaceae* | *Pseudomonas* |
| Fungi | Ascomycota | Dothideomycetes | Botryosphaeriales | *Botryosphaeriaceae* | *Botryosphaeria* |
|  |  |  |  |  | *Neofusicoccum* |
|  |  |  |  | *Phyllostictaceae* | *Phyllosticta* |
|  |  |  | Cladosporiales | *Cladosporiaceae* | *Cladosporium* |
|  |  |  | Mycosphaerellales | *Mycosphaerellaceae* | *Cercospora* |
|  |  |  |  |  | *Pallidocercospora* |
|  |  |  |  |  | *Pseudocercospora* |
|  |  |  | Pleosporales | *Didymellaceae* | *Didymella* |
|  |  |  |  |  | *Epicoccum* |
|  |  |  |  |  | *Phoma* |
|  |  |  |  |  | *Paraconiothyrium* |
|  |  |  |  |  | *Pseudopithomyces* |
|  |  |  |  | *Leptosphaeriaceae* | *Leptosphaeria* |
|  |  |  |  | *Massarinaceae* | *Stagonospora* |
|  |  |  |  | *Pleosporaceae* | *Alternaria* |
|  |  |  |  | *-* | *Pleosporales* nfc. |
|  |  | Eurotiomycetes | Eurotiales | *Aspergillaceae* | *Penicillium* |
|  |  |  |  | *Trichocomaceae* | *Talaromyces* |
|  |  | Sordariomycetes | Calosphaeriales | *Coniochaetaceae* | *Coniochaeta* |
|  |  |  |  | *Pleurostomataceae* | *Pleurostoma* |
|  |  |  | Diaporthales | *Diaporthaceae* | *Diaporthe* |
|  |  |  |  | *Valsaceae* | *Cytospora* |
|  |  |  |  |  | *Phomopsis* |
|  |  |  | Glomerellales | *Glomerellaceae* | *Colletotrichum* |
|  |  |  | Hypocreales | *Bionectriaceae* | *Clonostachys* |
|  |  |  |  | *Hypocreaceae* | *Trichoderma* |
|  |  |  |  | *Nectriaceae* | *Fusarium* |
|  |  |  | Ophiostomatales | *-* | *Ophiostomataceae* nfc |
|  |  |  | Sordariales | *Chaetomiaceae* | *Chaetomium* |
|  |  |  |  | *Sordariaceae* | *Neurospora* |
|  |  |  | Xylariales | *Hypoxylaceae* | *Daldinia* |
|  |  |  |  |  | *Hypoxylon* |
|  |  |  |  | *Sporocadaceae* | *Neopestalotiopsis* |
|  |  |  |  |  | *Pestalotiopsis* |
|  |  |  |  |  | *Pseudopestalotiopsis* |
|  |  |  |  | *Xylariaceae* | *Amphirosellinia* |
|  |  |  |  |  | *Biscogniauxia* |
|  |  |  |  |  | *Nemania* |
|  |  |  |  |  | *Rosellinia* |
|  |  |  |  |  | *Xylaria* |
|  |  |  |  |  | *Xylariaceae* nfc |
|  | Basidiomycota | Agaricomycetes | Hymenochaetales | *Hymenochaetaceae* | *Phellinus* |
|  |  |  | Russulales | *Peniophoraceae* | *Peniophora* |
|  |  | Malasseziomycetes | Malasseziales | *Malasseziaceae* | *Malassezia* |
|  |  | Microbotryomycetes | Sporidiobolales | *Sporidiobolaceae* | *Rhodosporidiobolus* |
|  |  |  |  |  | *Rhodotorula* |
